# Effect of a low-protein diet supplemented with keto-acids on autophagy and inflammation in 5/6 nephrectomized rats

**DOI:** 10.1042/BSR20150069

**Published:** 2015-10-30

**Authors:** Yue-yue Zhang, Juan Huang, Man Yang, Li-jie Gu, Jia-yao Ji, Li-jun Wang, Wei-jie Yuan

**Affiliations:** * Department of Nephrology, Shanghai General Hospital, Shanghai Jiaotong University School of Medcine, Shanghai 200080, China

**Keywords:** autophagy, chronic kidney disease, inflammation, ketoacids, muscle atrophy

## Abstract

Ketoacids (KA) are known to preserve muscle mass among patients with chronic kidney disease (CKD) on a low-protein diet (LPD). The present study was to compare the effects of KA supplemented diet therapy in autophagy and inflammation in CKD rats' skeletal muscle. Rats with 5/6 nephrectomy were randomly divided into three groups and fed with either 11 g/kg/day protein [normal-protein diet (NPD)], 3 g/kg/day protein (LPD) or 3 g/kg/day protein which including 5% protein plus 1% KA (LPD + KA) for 24 weeks. Sham-operated rats with NPD intake were used as control. LPD could improve body weight, gastrocnemius muscle mass, as well as gastrocnemius muscle cross-sectional area, with the effect being more obvious in the LPD + KA group. The autophagy marker LC3 (microtubule-associated protein 1 light chain 3), p62, Parkin and PTEN induced putative kinase 1 (PINK1) were significantly attenuate in LPD + KA group than LPD group. LPD + KA group had the lower total mtDNA (mitochondiral DNA) and cytosol mtDNA, NACHT-PYD-containing protein 3 (NALP3) inflammasome than LPD group, but its reactive oxygen species (ROS), caspase-1 and apoptosis-associated speck-like protein containing a CARD (ASC) level was higher. Immunoblotting showed IL-1β (interleukin-1-beta) was lower in LPD and LPD + KA group than the NPD group, but IL-18 showed no significant difference among control and CKD group; toll-like receptor signalling-dependent IL-6 was higher in LPD + KA group than LPD group, but tumor necrosis factor-α (TNF-α) was not significantly changed between LPD + KA and LPD group. Systematic changes of the four cytokines were different from that of the tissue. Although LPD + KA could further ameliorate-activated autophagy than LPD, its effect on the activated inflammation state in CKD was not distinctly. Further study is still required to explore the method of ameliorating inflammation to provide new therapeutic approaches for CKD protein energy wasting (PEW).

## Abstract (precis)

The present study demonstrated that autophagy/mitophagy was increased and inflammation was aggravated in skeletal muscle in chronic kidney disease (CKD) rats. A low-protein diet (LPD) supplemented with ketoacids (KA) improved the loss in muscle mass and blocked the activation of autophagy/mitophagy and inflammation in those rats.

## INTRODUCTION

The number of chronic kidney disease (CKD) patients has been constantly increasing around the globe. As long-term survival of CKD patients has been greatly improved thanks to the development of dialysis techniques, malnutrition is more prevalent and serious than ever before. Malnutrition and inflammatory state positively promote each other in a circle that accelerates arterial lesions and finally results in the malnutrition-inflammation-atherosclerosis syndrome; increasing mortality. Protein-energy wasting (PEW) was proposed to denote concurrent losses in protein and energy stores [1] and skeletal muscle wasting was the main characteristic of PEW. Identifying the mechanism of PEW and exploring interventions for it will help to provide practical and effective treatment approaches for improving the clinical prognosis of CKD patients.

Currently, it is believed that protein degradation resulting from chronic complications of CKD (e.g. metabolic acidosis, insulin resistance and micro-inflammatory state) has overtaken protein synthesis as the leading characteristic of skeletal muscle wasting. Besides the ubiquitin-proteasome system, the autophagy-lysosomal system presents an alternative system of degradation in eukaryotic cells. The primary role of autophagy (here means macrophagy), a highly conserved homoeostatic process, is to protect cells under stressful conditions, such as starvation and maintaining the amino acid pool essential for survival. However, if autophagy is excessively induced, it can result in pathological changes including cell death and apoptosis. Previously, the activation of autophagy has been demonstrated in skeletal muscle in a variety of conditions and in disease states ranging from fasting [2,3], oxidative stress [4], denervation [5,6] and drug effects [7,8] to some systemic diseases such as sepsis [9], laminin-deficient congenital muscular dystrophy (MDC1A) [10] and cancer [11]. In a previous study, this research team reported the up-regulation of mRNA and protein expression of microtubule-associated protein 1 light chain 3 (LC3), Gabarapl1 and Cathepsin L in the skeletal muscle of diabetic complicated with uraemia rats induced by subtotal nephrectomy [12].

Mitophagy, a kind of specific mitochondria-degrading autophagy, is capable of clearing abnormal mitochondria and controlling mitochondrial mass. In previous studies, this research team discovered that CKD could result in activation of mitophagy and increased mitochondiral DNA (mtDNA) levels in skeletal muscle [13]; consistently, we found this time that activated mitophagy is not capable of clearing abnormal mitochondria which aggravate skeletal muscle wasting through activation of inflammasome mediated by mtDNA and reactive oxygen species (ROS).

CKD is currently considered a low-grade inflammatory disease that can further promote protein wasting in skeletal muscle [14]. Moreover, increased proteolytic activity of caspase-1 in skeletal muscle by tumour-induced cachexia has been reported [15]. In addition, Rawat et al. [16] reported activation of NALP3 inflammasome in dyferlin-deficient skeletal muscle and proved that, in addition to immune cells, skeletal muscle cells can also form inflammasome when stimulated under certain conditions. Activation of NACHT-PYD-containing protein 3 (NALP3) could recruitment apoptosis-associated speck-like protein containing a CARD (ASC) and caspase-1, leading to the cascade of inflammation.

It is well known that protein-restricted diet therapy, as one of the main therapeutic strategies for CKD, can reduce glomerular hyper-transfusion and hyper-filtration [17]; whereas α-ketoacid (KA) supplemented low-protein diet (LPD) therapy can delay progression of renal disease and maintain nutritional status in CKD patients [18]. Previous studies from this research team found in the Ketosteril Research Award 2010 Foundation Project that, compared with normal-protein diet (NPD) therapy and LPD therapy, LPD+KA therapy could relieve skeletal muscle wasting in rats with diabetic nephropathy and improve the morphological and functional abnormalities of mitochondria [13]. In addition, it was also shown that rats treated with LPD+KA therapy had a lower level of autophagy in skeletal muscle than those in the normal protein and LPD groups [12]. These findings indicated that LPD+KA therapy relieves skeletal muscle wasting, most probably by reducing levels of activated mitophagy.

In previous studies by this research team, it has been shown that LPD+KA therapy can inhibit chronic inflammatory reaction in kidney tissues [19], but there are currently no reports of its effects on the chronic inflammatory state of skeletal muscle in CKD or that LPD+KA therapy could improve mitochondrial abnormality and resolve autophagy up-regulation and skeletal muscle wasting, inferring that mitophagy, as an adaptive protection mechanism, is capable of reducing inflammasome activation and consequently resolve skeletal muscle wasting by removing and clearing abnormal mitochondria. LPD+KA therapy can resolve skeletal muscle wasting by improving the abnormal mitophagy-inflammasome pathway.

Therefore, by using rats with 5/6 nephrectomy as the chronic renal insufficiency model, the present study was intended to observe changes in mitophagy, mtDNA, ROS, inflammasome and other inflammatory factors in skeletal muscle in CKD, to compare the effects of LPD+KA therapy in improving such changes and explain the molecular mechanism of LPD+KA therapy for improving skeletal muscle wasting in CKD, thus providing experimental evidence for clinical application of LPD+KA therapy in the treatment of CKD patients.

## MATERIALS AND METHODS

### Animals and experimental design

All the experiments were approved by the Animal Care and Use Committee of Shanghai Jiao Tong University and were performed in accordance with the National Institute of Health Guide for the Care and Use of Laboratory Animals. Male Sprague–Dawley rats (200–250 g), purchased from the Chinese Academy of Sciences, Shanghai, China, were maintained in cages at 22°C under a 12-h light/12-h dark cycle and were allowed free access to water. The animals were randomly assigned to either the 5/6 nephrectomy group or the sham-operated control group. Surgery was performed according to a previous method [20]. Briefly, after anaesthetized with pentobarbital, each animal in the nephrectomy group underwent 5/6 nephrectomy by surgical resection of the upper and lower thirds of the left kidney followed by right nephrectomy 7 days later. In the sham-operated rats, a sham operation was performed. On day 7 after the operation, the 5/6 nephrectomy group was then randomly divided into three groups maintained on three different diets: normal-protein diet (NPD; 11 g/kg/day protein, *n*=15), a LPD (3 g/kg/day protein, *n*=15) or a LPD supplemented with KA (3 g/kg/day protein, including 5% protein and 1% KA; LPD + KA, *n*=15). The sham group with the intake of the NPD acted as the control (*n*=10). KA (compound α-ketoacid) were provided by Fresenius-Kabi. All three diets were modified from AIN-93 (American Institute of Nutrition Rodent Diets) and contained the same calorie content [3.8 kcal/g (1 kcal ≡ 4184 J)] and the same vitamins and minerals. Study diets lasted for 24 weeks; at the end of the study, nine NPD rats, two LPD rats and two LPD + KA rats had died, but all controls survived.

### Blood and urine examination

The 24 h urine samples were collected in metabolism cages for protein analysis. Blood samples were collected after the animals received anaesthesia for the measurement of the serum albumin, creatinine and urea nitrogen levels. All assays were performed according to routine procedures in the Biochemical Laboratory of the Shanghai Jiaotong University Affiliated First People's Hospital.

### ELISA

Cytokine concentrations were determined by ELISA. The plasma levels of interleukin tumor necrosis factor-α (TNF-α), interleukin-6 (IL-6), IL-1β and IL-18 were analysed according to manufacturer's instructions. Hundred microlitres of each serum was mixed with assay buffer and absorbance was measured at 420 nm using a Synergy 2 ELISA plate reader (Bio-Tek).

### Histology

Gastrocnemius muscle samples were collected after the animals were killed. Samples were divided into three groups for either electron microscope(EM), Haematoxylin and Eosin (H&E) staining or were immediately frozen and kept at–80°C for real-time PCR and western blotting analyses.

### Transmission electron microscope (TEM)

For observation by TEM, muscle samples were fixed in 2% glutaraldehyde in 0.1 M cacodylate buffer. Specimens were post-fixed in 1% osmium tetroxide in the same buffer, dehydrated with graded series of ethanol and embedded in epon, as previously described [21]. Ultrathin sections were stained with uranyl acetate and lead citrated and were analysed by a Philips CM120 TEM (FEI).

### Haematoxylin and Eosin

Muscle samples were cleaned of any visible connective and adipose tissue and blood and then weighed. The gastrocnemius muscles were freeze-fixed in liquid-nitrogen-cooled isopentane and stored at–70°C, as previously described [22]. Serial transverse sections of 10 mm thickness were stained with H&E. The myofibre sizes were measured using Image-Pro Plus software (Media Cybernetics) and the muscle fibre cross sectional area (CSA) was calculated via the analysis of 50 myofibres of a muscle from each rat. The fibre CSA was determined from five areas at 40× magnification.

### Mitochondrial membrane potential

Mitochondrial membrane potential (MMP) was determined using the JC-1 (5,5′,6,6′-tetrachloro-1,1′,3,3′-tetraethylbenzimidazolcarbocyanine iodide) MMP detection kit (Beyotime). JC-1 is a cationic fluorescent dye probe (green as monomer/red as aggregates) which accumulates in mitochondria in a potential-dependent manner. Cells with functional mitochondria incorporate JC-1 leading to the formation of JC-1 aggregates, which show a red spectral shift resulting in higher levels of red fluorescence emission measured in the red (fluorescence, FL-2 channel) and green monomers (detectable in FL-1 channel). Cells with collapsed mitochondria contain mainly green JC-1 monomers. These were performed as previous described [23]. All of the standards, controls and samples were read in duplicate.

### Western blot analysis

Tissue lysates were prepared from samples frozen in liquid nitrogen. The samples were pulverized and lysed in Radio Immunoprecipitation Assay (RIPA) buffer for 2 h at 4°C. Lysates were centrifuged at 10000 ***g*** for 10 min at 4°C and the supernatants were transferred into separate tubes. Equal volumes (20 mg) of protein were separated using SDS/PAGE and transferred on to nitrocellulose membranes. The membranes were incubated overnight at 4°C in 5% skim milk with primary antibodies. Antibodies used were: PTEN induced putative kinase 1 (PINK1) (Abcam); Parkin (Abcam); LC3 (Abcam); P62 (Abcam); TNF-α (Abcam); IL-6 (Abcam); NALP3 (Abcam); IL-18 (Abcam); ASC (Santa); caspase-1 (Santa); IL-1β (Santa); GAPDH (glyceraldehyde-3-phosphate dehydrogenase; Cell Signaling Technology). Membranes were then washed and incubated using a secondary anti-rabbit IgG (Beyotime Institute of Biotechnology), antibody or anti-mouse IgG (Beyotime Institute of Biotechnology) or antibody conjugated with horseradish peroxidase (Beyotime Institute of Biotechnology). Band visualization was performed using an ECL Western Blotting Substrate kit (Millipore).

### ROS

Mitochondrial ROS was measured by MitoSOX (Invitrogen) staining (5 μM for 15 min at 37°C). To measure mitochondrial mass, tissues were stained with 25 nM of MitoTracker Green FM and MitoTracker Deep Red FM (Invitrogen; 15 min at 37°C). Data were acquired with a BD FACS Canto II flow cytometer (BD Biosciences) and analysed with FlowJo analytical software (Treestar).

### Quantitative real time PCR

Total genomic DNA was isolated from muscle samples with the TIANamp Genomic DNA kit (Tiangen) according to the instructions from the manufacturer. Relative mitochondrial DNA level was measured by performing quantitative real-time PCR performed in an ABI PRISM7300 Sequence Detection System. Two independent reactions were performed using established primers for mitochondrial and nuclear genes. mtDNA copy number was measured by quantitative PCR and normalized to nuclear DNA levels in a ratio of cytochrome *c* oxidase 1 (mtCOI) DNA over nuclear DNA (encoding 18S ribosomal RNA) [24]. The following primers were used:

18S forward, 5′-GAGAAACGGCTACCACATCC-3′;
18S reverse, 5′-CACCAGACTTGCCCTCCA-3′;
and COI forward, 5′-CATCCTCCATAGTAGAAGC-3′;
COI reverse, 5′-CCTAAGATAGAAGACACCC-3′.

For measurement of mtDNA in cytosol, the protein concentration and volume of the supernatant was normalized, followed by centrifugation at 10000 ***g*** for 30 min at 4°C to produce a supernatant corresponding to the cytosolic fraction. DNA was isolated from 200 μl of the cytosolic fraction. Copy number of mtCOI DNA was measured by quantitative real time PCR using same volume of the DNA solution.

### Statistical analysis

SPSS 17.0 (SPSS) was used for statistical analysis. For data that were normally distributed, one-way ANOVA was implemented followed by pairwise comparison by the least significant difference test. When the experimental groups were unequal and non-parametric, data were analysed by ANOVA on ranks and then by Dunn's method for pairwise comparison. Data are expressed as means and S.D.s. *P*<0.05 was considered as statistically significant.

## RESULTS AND DISCUSSION

PEW of CKD is closely associated with major adverse clinical outcomes and results with increased rates in hospitalization and death in these patients, especially with end-stage renal disease and maintenance dialysis [25]. Approximately 70%–75% of the patients with end-stage renal disease were found to have PEW [26]. For decades, protein restriction has been used to alleviate uremic symptoms, to protect the function of remnant kidneys, as well as improve complications such as abnormal glucose metabolism and hypertension in patients with CKD [27–29]. In studies on CKD, several reports have demonstrated that a LPD prevented the progression of renal injury in both humans and animals [30].With sufficient energy intake and careful monitoring for dietary compliance, a LPD is nutritionally safe in patients with early CKD. KA capture excess nitrogen residues and utilize these residues for essential amino acid production. Thus, nitrogen intake may be restricted and endogenous urea formation reduced. Furthermore, if there is a sufficient amount of essential amino acids, an accumulation of non-excreted, potentially toxic ions and metabolic products arising from the breakdown of foods rich in protein may be avoided [31]. Several studies report that the keto-diet could also slow the rate of decline in renal function, with better outcomes after the initiation of dialysis [32–34]. Results of a single-centre randomized controlled trial addressing the rate of CKD progression revealed a 57% slower decline in renal function with the keto-diet compared with a conventional LPD. The keto-diet allowed the safe management of selected patients with stages 4–5 CKD, delaying dialysis for almost 1 year, with a major impact on patient quality of life and health expenditures [35]. Previous animal studies found that the addition of KA to an LPD prevents weight loss and completely normalizes serum albumin levels, indicating that LPD supplemented with KA can maintain nutritional status [36], ameliorating protein malnutrition and oxidative stress injury in remnant kidney tissue [37].

### Ketoacid treatment could further improve body weight and gastrocnemius muscle atrophy caused by CKD than LPD

A low body mass index is associated with a poor outcome and a high risk of death [38]. The present study found that the body weights and gastrocnemius muscle mass of rats from the CKD groups were significantly lower than those of the control group. Among the CKD groups, the body weights and gastrocnemius muscle mass in the NPD group were the lowest, which was higher in LPD + KA group than LPD group, but the difference was not significant. The cross-sectional area of muscle fibre was considered as the best indicator for muscle atrophy, thus avoiding potential confounding factors related to changes in extracellular space. The mean cross-sectional area of NPD group was 56% lower than the control group. Fibre atrophy was attenuated in the LPD and LPD + KA group. LPD + KA group was higher than LPD group, but the difference was not significant (Figure 1).

**Figure 1:**
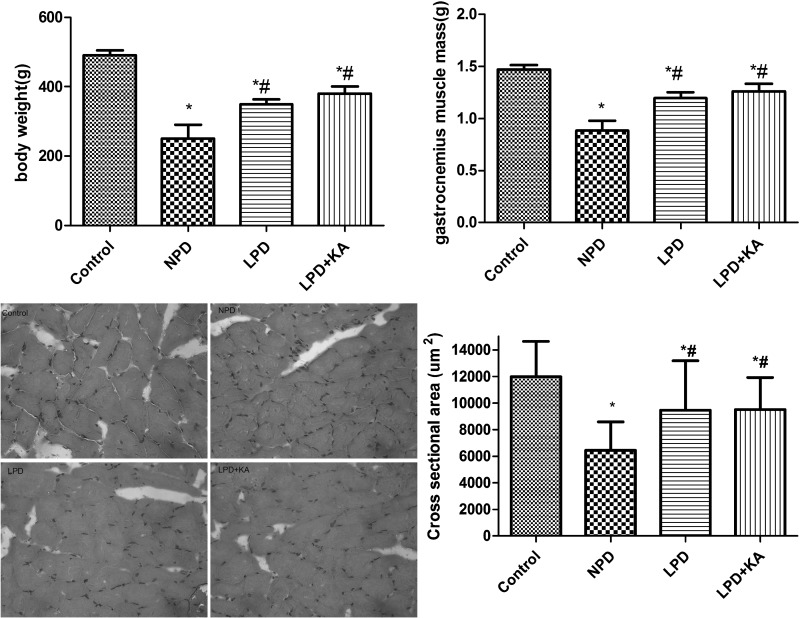
Body weights and gastrocnemius muscle mass of the experimental groups *Compared with control group, *P*<0.05. #Compared with NPD group, *P*<0.05. Abbreviation: Control, sham group with NPD.

### Ketoacid treatment could further improve urinary protein and biochemical parameters

Serum albumin, a biochemical indicator in the diagnosis of PEW, is a strong predictor of mortality in maintenance haemodialysis patients [39]. Serum albumin was lower in the CKD groups than the control group. Among the CKD groups, the LPD group was lower than NPD group, LPD + KA group was higher than the LPD group, but differences were not significant. Blood urea nitrogen and creatinine were highest in the NPD group and reduced in the LPD group; the LPD + KA group had the lowest values, but the difference was not significant among CKD groups. Urinary protein was found to be highest in the NPD group, significantly decreased in the LPD group and lowest in the LPD + KA group (Figure 2).

**Figure 2:**
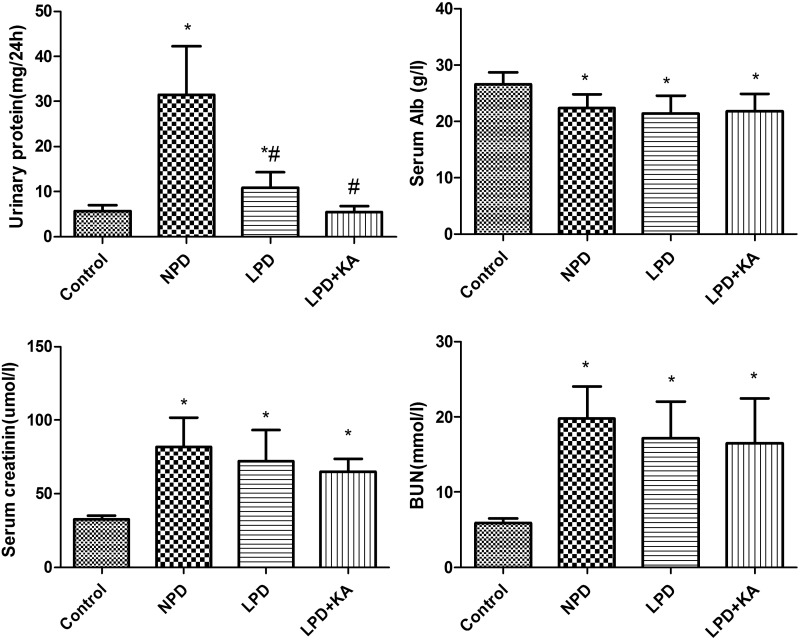
Urinary protein and biochemical parameters of the experiment groups Urinary protein, serum, serum creatinine, blood urea nitrogen of Control, NPD, LPD and LPD + KA group. Data are expressed as mean ± S.D. *Compared with control group, *P*<0.05. #Compared with NPD group, *P*<0.05. Abbreviations: Alb, albumin; BUN, blood urea nitrogen; Control, sham group with NPD; sCr, serum creatinine.

### Ketoacid treatment could further improve autophagy/mitophagy in gastrocnemius muscle

In autophagy–lysosomal pathway, p62 is an ubiquitin-binding scaffold protein that directly binds to LC3 to facilitate ubiquitinated protein aggregates degradation. Immunoblotting showed that p62 and LC3 increased in the CKD groups, among which, p62 and LC3 were distinctly higher in the NPD group than the control group, but were lower in LPD group, although p62 did not change significantly. In addition, KA supplementation further reduced the p62 and LC3, but the difference was also not significant (Figures 3A–3C).

**Figure 3:**
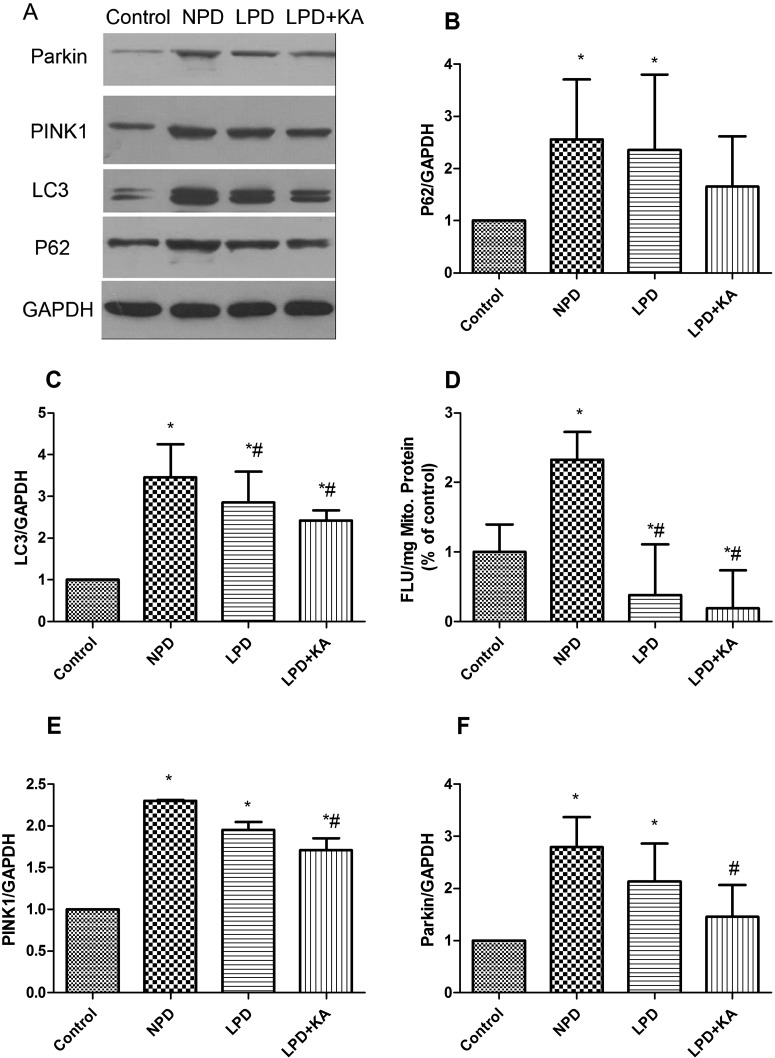
Change of autophagy/mitophagy in the experimental groups (**A**) Representative immunoblotting of P62, LC3, PINK1, Parkin and GAPDH. The ratio of P62–GAPDH (**B**); LC3–GAPDH (**C**); PINK1–GAPDH (**E**); Parkin–GAPDH (**F**) normalized to the control group of Control, NPD, LPD and LPD + KA group. (**D**) Decrease in MMP of the four groups presented as fluorescent JC-1 dye (FLU 6 SEM/mgP). *Compared with control group, *P*<0.05. #Compared with NPD group, *P*<0.05. &Compared with LPD group, *P*<0.05. Abbreviation: Control, sham group with NPD.

Mitochondria depolarization, a decrease in MMP, is a marker of the initiation of mitophagy. The NPD group showed significant decrease in MMP than the control group, LPD could attenuate the decrease in MMP, whereas LPD + KA could further decrease MMP (Figure 3D). As is known, mitochondrial-localized PINK1, which is normally undergoes rapid degradation, is stabilized on the outer mitochondrial membrane (OMM) when mitochondria are depolarized [40]. Accumulated PINK1 recruits cytosolic Parkin on to depolarized mitochondria, resulting in activation of its E3 activity and Parkin then ubiquitinates mitochondrial substrate. The phosphorylation of substrates by PINK1 primes protein of OMM to either interact with Parkin, thus sequestering cytosolic Parkin to the mitochondria or to be ubiquitinated by Parkin [41]. Parkin and PINK1 were highest in the NPD group and decreased in the LPD group, although the difference was not significant. KA supplementation further reduced Parkin and PINK1 and the difference between NPD group and LPD + KA group was significant (Figures 3E and 3F).

As to the TEM images, we found distinctly more autophagosome or autolysosome in NPD group than control group, but in the LPD group the autophagosome or autolysosome were significantly decreased and further decreased in the LPD + KA group, with the LPD + KA group similar to the control group (Figure 4).

**Figure 4:**
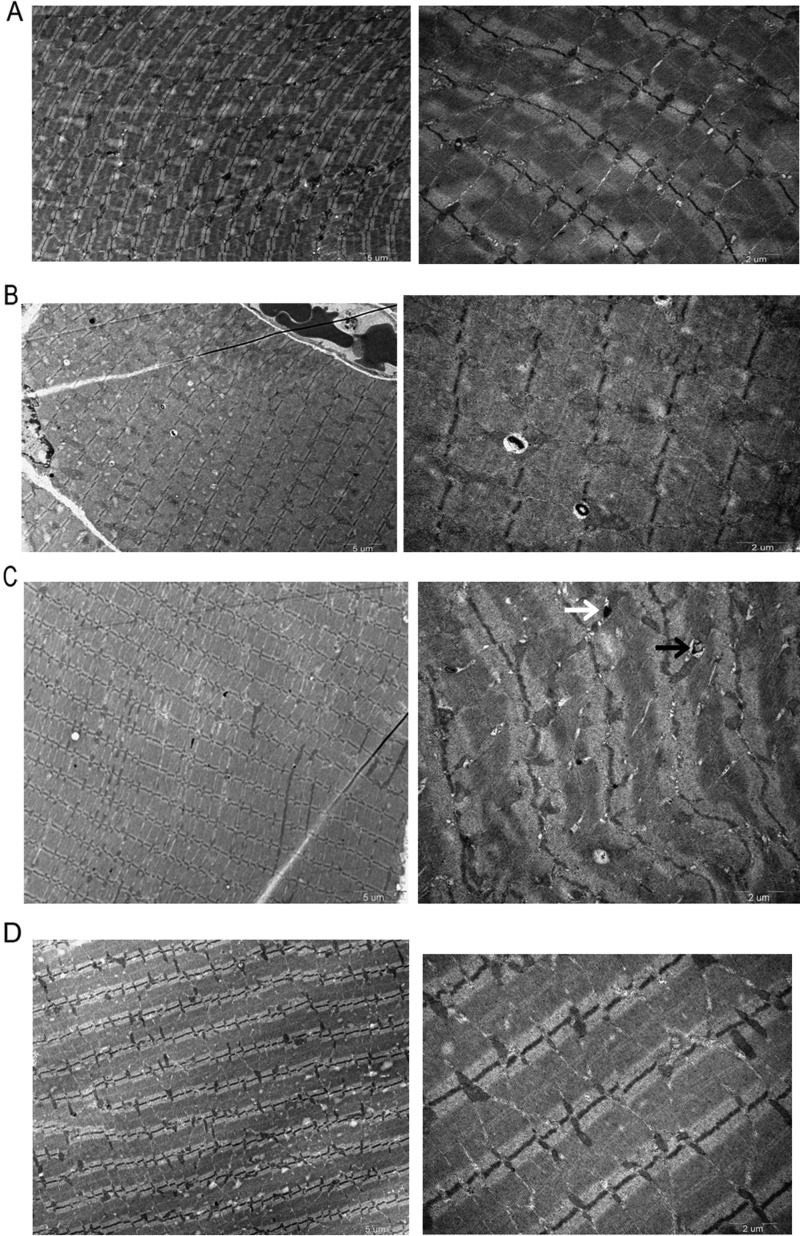
Representative electron micrograph of a section of gastrocnemius muscle in the experiment groups detected by TEM Autophagosomes (black arrows) or autolysosomes (white arrows). The autophagy of NPD was significantly higher than that of the other three groups, LPD + KA group was significantly more attenuated than LPD group. Abbreviation: Control, sham group with NPD.

### Effects of ketoacid treatment on inflammation state

Although insufficient food intake (true under-nutrition) due to poor appetite and dietary restrictions contributes to these problems, there are features of the syndrome that cannot be explained by under-nutrition alone. Many contributing causes are directly related to kidney disease, including increased resting energy expenditure, persistent inflammation, acidosis, multiple endocrine disorders and the dialysis procedure itself and muscle wasting is considered one of the most valid markers of PEW in CKD, associated with inflammation and increased mortality [42].

As the generator of ATP and the main site that produces ROS, mitochondria are easily damaged due to change of environment inside and outside cells. In addition, abnormal mitochondria lead to cell injury through a disorder of energy metabolism, oxidation disorder, ROS and mtDNA released to cytosol. Recent research highlighted that mitochondria-produced ROS could promote release of mtDNA into the cytoplasm [24], and activation of inflammasome to mediate inflammatory reactions [43]. In the present study, total mtDNA and mtDNA released into cytosol was higher in the NPD group compared with control group, LPD group was lower than the NPD group, whereas KA supplement further reduced mtDNA (Figure 5). ROS was higher in the NPD group than control group, the LPD group was lower than the NPD group, whereas KA supplement increased ROS again and the difference was significant between the LPD + KA and the LPD groups, but remained significantly lower than the NPD group (Figure 6). We further studied NALP3 inflammasome and caspase-1 and found they were higher in the NPD group compared with control group, the LPD group was lower than the NPD group, whereas KA supplement further reduced NALP3 inflammasome but not caspase-1; but ASC showed a slight difference (Figures 7A–7C). ASC was lower in NPD group and further decreased in LPD group, but distinctly increased in LPD + KA group (Figure 7D).

**Figure 5:**
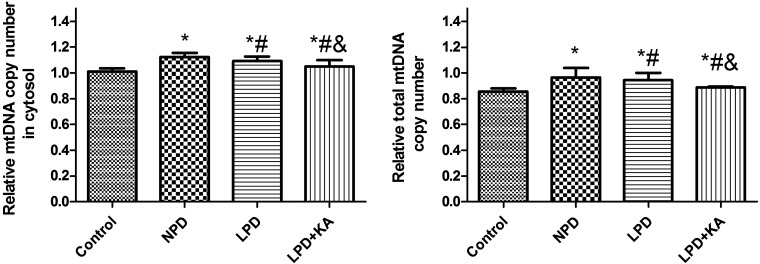
CT value of mtDNA of Control, NPD, LPD and LPD+KA group normalized to control group Abbreviation: Control, sham group with NPD.

**Figure 6:**
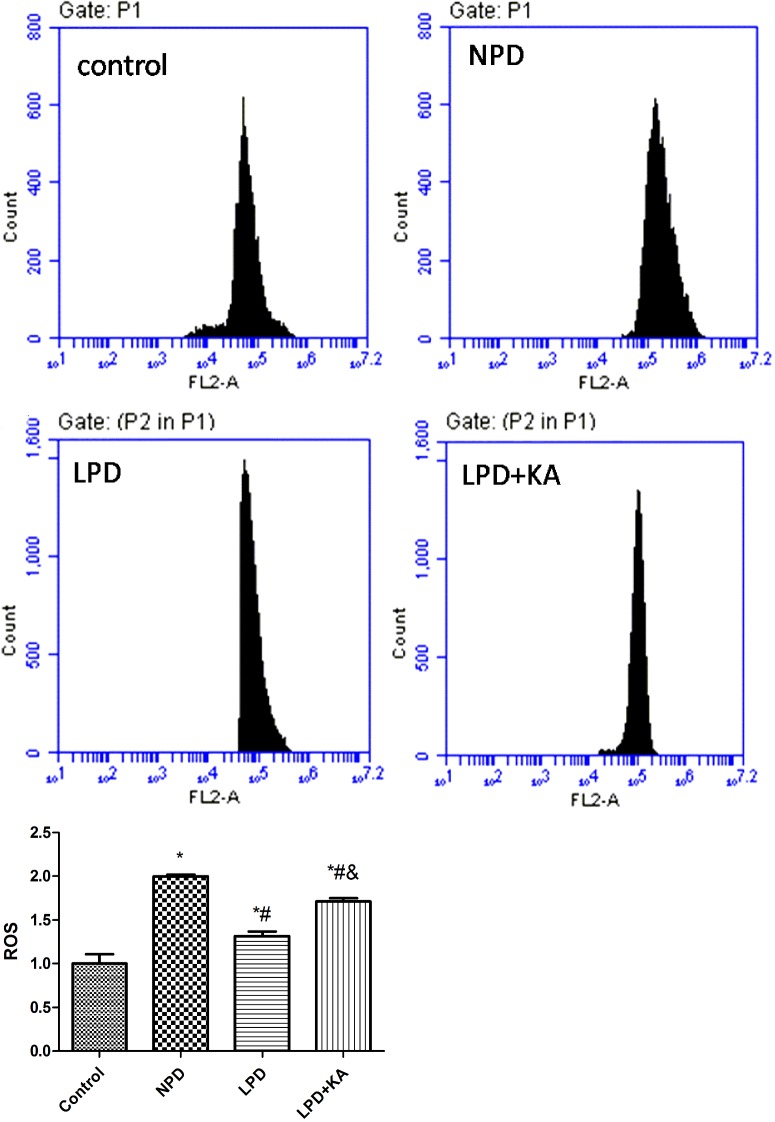
ROS of Control, NPD, LPD, LPD + KA group detected by mitoSOX Representative image of Control (**A**), NPD (**B**), LPD (**C**) and LPD + KA (**D**) group. (**E**) Average of ROS of each group. *Compared with control group, *P*<0.05. #Compared with NPD group, *P*<0.05. &Compared with LPD group, *P*<0.05. Abbreviation: Control, sham group with NPD.

**Figure 7:**
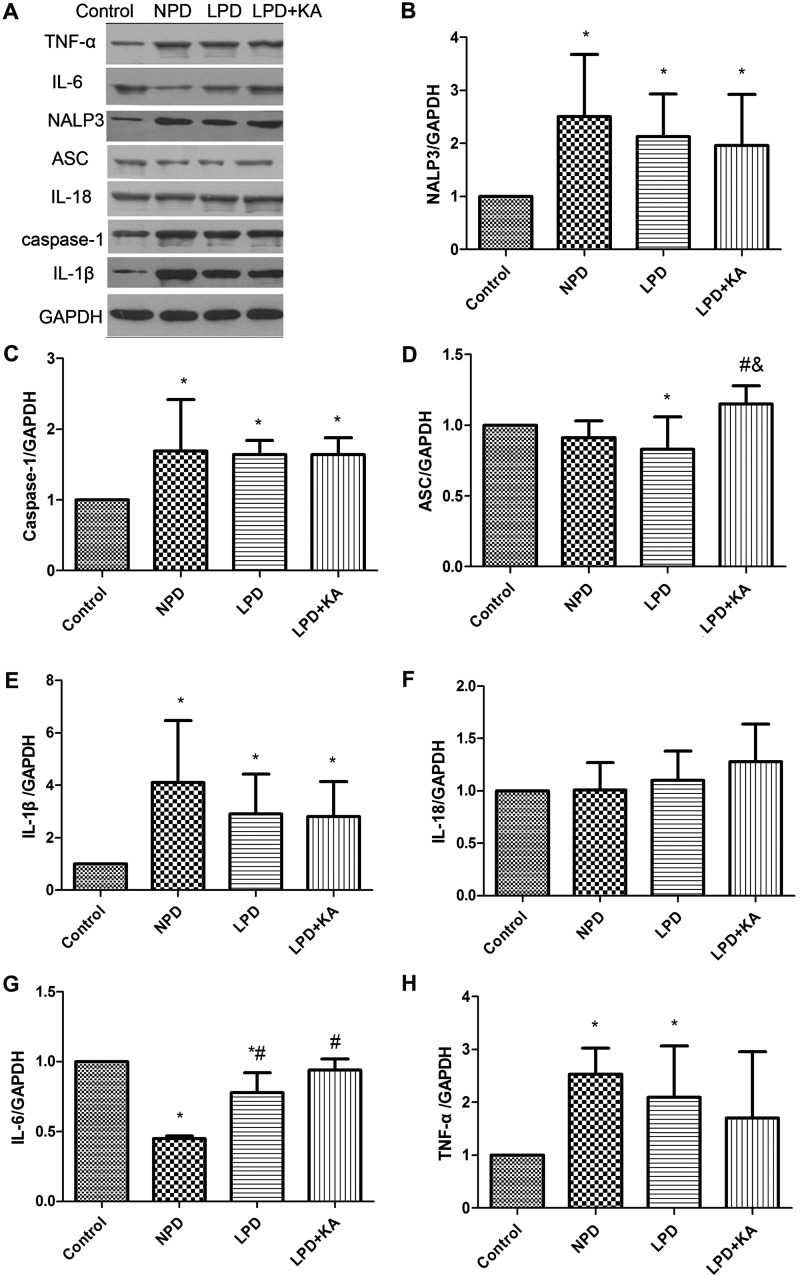
Change of inflammatory factor in the experimental groups detected by immunoblotting (**A**) Representative immunoblotting of IL-1β, IL-18, IL-6, TNF-α, NALP3, ASC, caspase-1 and GAPDH of Control, NPD, LPD and LPD + KA group. The ratio of NALP3–GAPDH (**B**); caspase-1–GAPDH (**C**); ASC–GAPDH (**D**); IL-1β–GAPDH (**E**); IL-18–GAPDH (**F**); IL-6–GAPDH (**G**); TNF-α–GAPDH (**H**) normalized to the control group of Control, NPD, LPD, LPD + KA group. *Compared with control group, *P*<0.05. #Compared with NPD group, *P*<0.05. &Compared with LPD group, *P*<0.05. Abbreviation: Control, sham group with NPD.

Inflammasome is a molecular platform for activation of caspase-1, which, when intracellular signals identify danger or microbial infection, becomes activated by binding with inflammasome to cleave such precursors of inflammatory factors as pro-IL-1β and pro-IL-18 resulting in secretion of ‘mature’ IL-1β and IL-18, consequently promoting the inflammatory cascade [43]. In the present study, immunoblotting showed IL-1β was higher in the NPD group, was lower in LPD and LPD + KA than the NPD group; IL-18 gradually increased among control, NPD, LPD and LPD + KA group, but there's no significant difference(Figures 7E and 7F); the secretion of IL-6 or TNF-α, which depends on toll-like receptor signalling, were not similar with that of IL-1β and IL-18 (Figures 7G and 7H). IL-6 was lowest in NPD group, higher in LPD + KA group than LPD group, but the change was not significant. TNF-α was highest in NPD group, lower in LPD + KA group than LPD group, but the change was not significant. Systematic changes were slightly different from those seen in tissue as shown by the ELISA results: IL-1β was highest in NPD group, LPD + KA group was a little higher than the LPD group, but the difference was not significant. IL-18 was highest in NPD group, was a little lower in LPD + KA group than the LPD group, but the difference was not significant (Figures 8A and 8B). Meanwhile, IL-6 and TNF-α did not have a similar change, they gradually increased among control, NPD, LPD and LPD + KA group, but there's no significant difference between CKD groups (Figures 8C and 8D).

**Figure 8:**
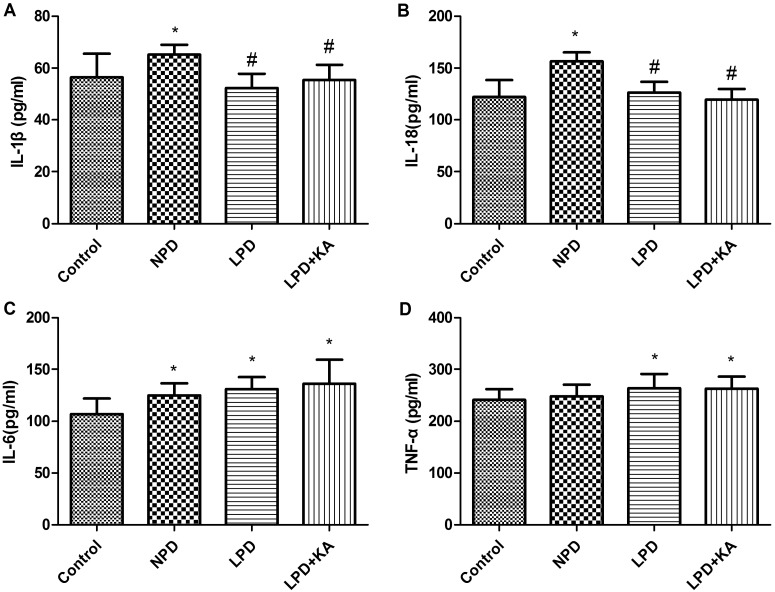
Change of inflammatory factor in the experimental groups detected by ELISA IL-1β (**A**); IL-18 (**B**); IL-6 (**C**); TNF-α(**D**) of Control, NPD, LPD and LPD + KA group. *Compared with control group, *P*<0.05. #Compared with NPD group, *P*<0.05. &Compared with LPD group, *P*<0.05. Abbreviation: Control, sham group with NPD.

In summary, the present study demonstrated that autophagy/mitophagy was increased and inflammation was aggravated in skeletal muscle in CKD rats. In addition, a LPD supplemented with KA improved the loss in muscle mass and blocked the activation of autophagy/mitophagy and inflammation in the skeletal muscle of CKD rats. Thus, these findings may provide relevant preclinical data for the use of a LPD supplemented with KA in patients with CKD. Besides inflammation, acidosis could also lead to muscle atrophy, Marino et al. [44] found that acute acidic stress stimulates a protective autophagic response in melanoma cells, which may also be a new therapy perspective to CKD muscle atrophy.

## Abbreviations

ASC: apoptosis-associated speck-like protein containing a CARD
CKD: chronic kidney disease
GAPDH: glyceraldehyde-3-phosphate dehydrogenase
H&E: Haematoxylin and Eosin
I L-6: interleukin-6
KA: ketoacids
LC3: microtubule-associated protein 1 light chain 3
LPD: low-protein diet
MDC1A: laminin-deficient congenital muscular dystrophy
MMP: mitochondrial membrane potential
mtCOI: cytochrome *c* oxidase 1
mtDNA: mitochondiral DNA
NALP3: NACHT-PYD-containing protein 3
NPD: normal-protein diet
OMM: outer mitochondrial membrane
PEW: protein-energy wasting
PTEN: induced putative kinase 1
ROS: reactive oxygen species
TNF: tumor necrosis factor
